# Enhancing the Efficacy of Tumor Vaccines Based on Immune Evasion Mechanisms

**DOI:** 10.3389/fonc.2020.584367

**Published:** 2021-02-03

**Authors:** Jianyu Chen, Honghao Zhang, Lijuan Zhou, Yuxing Hu, Meifang Li, Yanjie He, Yuhua Li

**Affiliations:** ^1^ Department of Hematology, Zhujiang Hospital, Southern Medical University, Guangzhou, China; ^2^ Bioland Laboratory (Guangzhou Regenerative Medicine and Health Guangdong Laboratory), Guangzhou, China

**Keywords:** tumor vaccines, immune evasion, tumor microenvironment, combination therapies, immunotherapy

## Abstract

Tumor vaccines aim to expand tumor-specific T cells and reactivate existing tumor-specific T cells that are in a dormant or unresponsive state. As such, there is growing interest in improving the durable anti-tumor activity of tumor vaccines. Failure of vaccine-activated T cells to protect against tumors is thought to be the result of the immune escape mechanisms of tumor cells and the intricate immunosuppressive tumor microenvironment. In this review, we discuss how tumor cells and the tumor microenvironment influence the effects of tumor infiltrating lymphocytes and summarize how to improve the efficacy of tumor vaccines by improving the design of current tumor vaccines and combining tumor vaccines with other therapies, such as metabolic therapy, immune checkpoint blockade immunotherapy and epigenetic therapy.

## Introduction

Immunotherapy aims to initiate or reinitiate the self-sustaining cycle of tumor immunity, and there is great expectation that this approach will cure various tumor types. Many tumor-targeting approaches exist, but few tumor therapies induce as durable activity as immunotherapy. Safe and robust tumor vaccines have held great promise for tumor immunotherapy.

Tumors are known for their accumulation of genetic alterations and loss of normal cellular regulatory processes (1). These events can lead to the expression of tumor antigens, resulting in peptides that bind to major histocompatibility class I (MHC-I) molecules on the surface of tumor cells (2). These tumor specific peptide-MHC-I complexes can be recognized by CD8^+^ T cells (3). The aim of tumor vaccines is to expand the tumor-specific T cell pool from the naïve pool and reactivate existing tumor-specific T cells in dormant or unresponsive states. Despite tremendous potential of tumor vaccines for tumor immunotherapy, the clinical outcomes in some patients remain suboptimal. In the past, this may be attributed to the selection of tumor antigen, as traditional tumor vaccines mainly target tumor-associated antigens (TAAs), which can be detected on both tumor cells and normal cells (4, 5). Currently, the development of sequencing technologies and different bioinformatics algorithms have accelerated the identification of neoantigens and the construction of neoantigen tumor vaccines (6, 7). Neoantigens are highly immunogenic because they are only expressed on tumor cells and do not present in normal cells, hence bypassing central thymic tolerance (4). Clinical trials have shown that neoantigen vaccine strategies successfully increase the frequency and activity of tumor-specific cytotoxic T lymphocytes (CTLs) (8, 9). However, tumor development is a dynamic progression. Owing to the immune suppression and escape mechanisms of tumor tissues, neoantigen vaccines alone may not be able to apply expected anti-tumor protection, even if host immune systems have been activated, for the reason that the vaccine alone fails to ensure that the activated T cells home to the tumor bed and exert anti-tumor effects within the tumor.

In fact, the immune response in tumors is precisely regulated in a cancer-immunity cycle. First, the tumor antigens should be released and presented by antigen presenting cells (APCs) to T cells through the T cell receptor (TCR) in order to prime and activate the tumor-specific effector T cells. Second, activated effector T cells must be trafficked to tumor bed and infiltrate into the tumors to specifically recognize the tumor cells. Last, the effector T cells kill the tumor cells (10). Tumor vaccines should go through the above immunity cycle to produce a tumor killing effect. Unfortunately, many tumor specific T cells have become victims of immune suppression and immune escape mechanisms, which means that the vaccine-activated T cells become exhausted or dysfunctional before they exert tumor-killing effects. This is one of the most important reasons for tumorigenesis, recurrence and metastasis in relation to both tumor cell-intrinsic factors and the tumor microenvironment (TME). In this review, we mainly focus on the key aspects of how tumor specific T cells are controlled by the tumor cells and tumor microenvironment and are manipulated to enhance the anti-tumor immunity of tumor vaccines to implement new clinical strategies.

## Modulation of T Cell Function by Tumors

**Figure 1:**
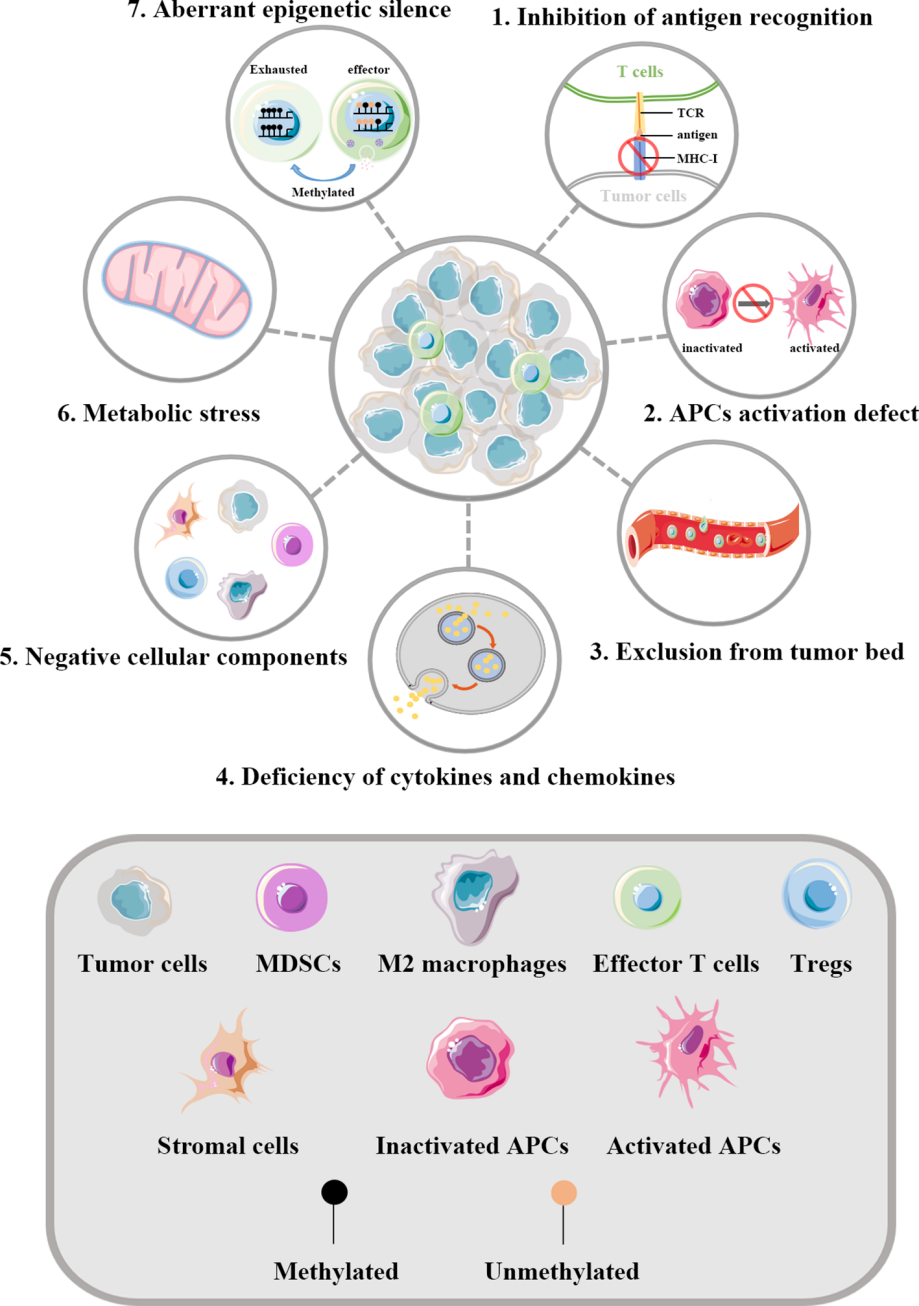
Regulation of T cells by tumors. The key aspects of how tumors influence the function of effector T cells are illustrated. 1) Abnormal alterations of MHC-I molecules in tumor cells can help evade the recognition of T cells. 2) A series of factors in TME can inhibit the maturation of APCs, thus defecting the priming and activating of effector T cells. 3) Tumor blood vessels act as physical barriers affecting the infiltration of T cells into the tumor bed. The vascular endothelial growth factors generated by tumor derived blood vessels can result in multiple changes within the vessels and thereby drive T cells depletion. 4) Abnormal cytokines and chemokines in TME can influence the infiltration of T cells. In tumors lacking in the infiltration of effector T cells, the expression of chemokines involved in the recruitment of T cells are significantly reduced. 5) Negative cellular components are key cellular mediators reshaping the immunosuppressive TME. 6) Dysregulated metabolism pathways in tumor cells can lead to insufficient nutrients in TME. What’s more, the specialized metabolism of tumor cells establishes an unfriendly TME to effector T cells, which further increases the living stress of effector T cells. 7) Effector T cells infiltrating in TME are frequently in exhaustion state and accumulating evidence implies such reprogramming to be the consequence of aberrant epigenomes such as methylated. TCR, T cell receptor; MHC-I, major histocompatibility class I molecules; APCs, antigen presenting cells; MDSCs, myeloid-derived suppressor cells; Tregs, regulatory T cells.

Tumor evasion immunity comprises both tumor cell-intrinsic alterations and TME modification. These components form a complex immunosuppressive network in tumors, which together limits the activation of and induces the dysfunction of T cells. **Figure 1** shows how tumors influence the function of effector T cells.

### Inhibition of Recognition, Priming, and Activation

Tumorigenesis is the process of continuous tumor cell evolution. A series of changes may occur in tumor cells to evade the recognition of immune cells. MHC-I molecules presented on the surface of tumor cells are key proteins for CD8^+^ T cell recognition. However, MHC-I molecule abnormalities have been found to occur at a relatively high frequency in tumor cells, including the loss or downregulation of MHC-I molecules (11, 12). This leads to the conclusion that alterations of MHC-I molecules stand for a common immune-escape mechanism of tumor cells (13). Actually, the expression of MHC-I molecules is the result of a proper antigen processing machinery (APM) and any alterations in the APM may lead to the deficiency of antigen processing and cause immune escape. For example, transporter associated with antigen processing (TAP) plays an important role in the transportation of proteins and the alteration of TAP can induce a sharp decrease of the MHC-I/b2m-peptide complexes expressed on tumor cells (14, 15). Indeed, the deficiency of TAP can be found in a variety of tumors such as head and neck carcinoma, gastric cancer and cervical carcinoma (14–17). Apart from the above gene regulatory mechanisms, the presentation of tumor antigens can be affected by other tumor cell biological processes. For example, the expression of TAP, latent membrane protein (LMP), Tapasin and MHC-I molecules are recovered after histone deacetylase inhibitor (HDACi) treatment, suggesting that epigenetic repression is also involved in the mechanism of tumor MHC-I molecules loss (18).

In addition to the intrinsic tumor cell factors, a series of factors in the TME, such as regulatory T cells (Tregs), disrupt the antigen presentation process, leading to insufficient T cell activation. Cross-presentation of tumor antigens by dendritic cells (DCs) is considered important in the early stage of tumor immune recognition because DCs acquire, process, and present tumor antigens to TCR and provide co-stimulatory factors to prime and boost the CD8^+^ T cells. However, CTLA-4 expressed on Tregs can suppress such immune response through the interaction with co-stimulation factors CD80 and CD86 expressed on DCs. When combined, the expression of CD80 and CD86 on DCs will be downregulated, thus impairing DC function and inhibiting T cell stimulation (19, 20). Moreover, a novel study uncovered that the antigen-specific Tregs activated by DCs can form a strong interaction with DCs. Such strong binding can remove the peptide-major histocompatibility complex class II (pMHCII) complexes from the surface of DCs and thereby decrease the antigen presentation efficiency of DCs (21). The upregulation of lymphocyte activation gene-3 (LAG-3) expression on activated Tregs is also a factor that acts as an immune regulatory protein (22).

Other components in TME may also influence the activation of effector T cells. Metabolic stress, including hypoxia and glucose deficiency, can cause downregulation of MHC-I molecules on tumor cells. Such alteration is accompanied by the loss of sensitivity of tumor cells to the upregulation of MHC molecules mediated by interferon (IFN)-γ (23). Programmed cell death 1 ligand (PD-L1) and Programmed cell death 2 ligand (PD-L2) expressed on tumor cells and other cells are also indispensable factors that inhibit proliferation and cytokine production of programmed cell death (PD)-1 expressing T cells (24).

### Inhibition of Trafficking and Infiltration to Tumor Bed

Chemokines and cytokines regulate the trafficking and infiltration of immune cells into the TME. Researchers have found that in tumors lacking in the infiltration of CD8^+^ T cells, the expression of chemokines involved in the recruitment of effector T cells was significantly reduced (25), implying their critical roles in tumor progression. On the one hand, chemokine expression is regulated by environmental cues in the TME. In transplanted melanoma mouse model, Barreira da Silva et al. showed that the dipeptidylpeptidase (DPP4) produced by stromal cells within the tumor inactivated chemokine CXCL10, leading to the reduction of T cell infiltration (26). On the other hand, chemokine expression can be regulated by tumor cell-intrinsic epigenetic and genetic mechanisms. Tumor epigenetic silencing often includes zeste homologue2 (EZH2)-mediated histone modifications and DNA methyltransferase 1 (DNMT1)-mediated DNA methylation. Using the ovarian cancer mouse models, researchers found repression of T helper 1 (Th1)-type chemokines CXCL9 and CXCL10 produced by tumor cells caused by the above epigenetic silencing. The finding indicates that there exists a negative association between tumor-infiltrating CD8^+^ T cells and epigenetic silencing (27). A similar change was found in C-C motif chemokine ligand (CCL)5, where DNA methylation reduced the expression of CCL5 and caused tumor-infiltration lymphocyte desertification (28). Moreover, using a genetically engineered mouse melanoma model, researchers found that oncogenic pathway is another approach displayed by tumor cells to control chemokine expression. In the melanoma model, the activation of β-catenin resulted in poor expression of CCL4, which is important for the migration of CD103^+^ DCs, and subsequently limited the activation and infiltration of effector T cells (29).

Another reason accounting for the defective T cell infiltration to the tumor sites could be the presence of physical barriers. Like normal tissues, tumors need to obtain nutrients and oxygen and excrete metabolic wastes to maintain their survival, and the generation of tumor-associated neo-vasculature can meet these needs. However, such blood vessels are produced under the condition of an unbalanced mix of proangiogenic signals (1) and can act as physical barriers to the transportation of T cells into the tumor bed. When T cells enter the tumor vessels, a series of changes in the tumor vasculature repress T cell activity and induce T cell exhaustion. First, intercellular adhesion molecule-1 (ICAM-1) and vascular cell adhesion molecule-1 (VCAM-1) are needed for the extravasation of T cells and upregulation of ICAM-1 has been associated with good prognosis of cancer patients (30–33). However, tumor-derived blood vessels can generate vascular endothelial growth factors (VEGF) to inhibit the expression of adhesion molecules and thus prevent T cells from infiltrating into the tumor (34). Second, as within the tumor, inhibitory regulatory molecules such as PD-L1, PD-L2, galectin-9, indoleamine 2,3-dioxygenase 1 (IDO-1), and human B7 homolog 3 (B7-H3) (35–39) can be upregulated on vascular endothelial cells to directly inhibit T cell activity. VEGF-α can also induce the expression of thymocyte selection-associated high mobility group box (TOX) protein in T cells, thereby driving T cell depletion (40). Third, Fas-L expressed on tumor endothelial cells can directly cause T cell apoptosis (41). Finally, tumor blood vessels are known for their unique characteristics, such as slow and irregular blood flow, microvascular disorders, lack of basement membranes and abnormally thick membranes in blood vessels (42), which together result in insufficient oxygen supply in blood vessels and the accumulation of metabolic waste, leading to the impairment of T cells. What’s more, these factors would trigger the release and activation of pro-angiogenic growth factors (43) and further deepen the damage to T cells, as demonstrated above.

### Impairment of T Cell Function and the Induction of Dysfunctional T Cells

Tumor cells rely on metabolic networks to maintain their proliferation, and various metabolism pathways are dysregulated in tumor cells due to their irregulated genetic landscape. Like tumor cells, having enough nutrients is essential for normal metabolic activity and anti-tumor immune response of T cells. However, the competition for nutrients between tumor cells and T cells can affect the immune response, and depletion of nutrients in the TME can also lead to insufficient immune response and tumor progression. Glucose is a major energy source, and it plays an important role in maintaining normal cellular functions and supporting cellular bioenergy. Even when oxygen is sufficient, tumor cells utilize glucose *via* glycolysis, which is called the Warburg effect (44). The glycolytic enzyme hexokinase 2 (HK2) overexpressed in tumor cells ensures the high efficiency of glycolysis in tumor cells and at the same time inhibits the glucose uptake of T cells (45, 46). Long-term glucose deficiency results in low T cell response and impairs the production of cytokines, allowing tumors to acquire immune escape properties. In addition to glucose, amino acids and lipids are also metabolic sources competed for by tumor cells and T cells. For example, IDO is expressed in many tumors and can catabolize tryptophan (47). Lower concentrations of tryptophan in extracellular environment can inhibit the proliferation of CD8^+^ T cells and promote the differentiation of Tregs by activating general control nonderepressible 2 (GCN2) kinase (48). Lipid rafts in the cell membrane of T cells are required to form immune synaptic tissues (49), while the growing tumor cells also need fatty acids to synthesize cell membranes or other molecules (50). The disturbance of lipid homeostasis may therefore result in a reduction of effector T cells.

In addition to nutrient depletion, the specialized metabolism of tumor cells also establishes a hypoxic, acidic TME (44) that is unfriendly to the anti-tumor immune response. In other words, besides promoting the growth of tumor cells, the unique metabolic programs can also prevent the development of an effective anti-tumor response. For example, the reduced blood flow and Warburg effect can result in a hypoxia state in the TME. Earlier studies have indicated that hypoxia can lead to the deficiency of mTOR signaling in T cells which can drive the anergy of effector T cells (51, 52) while promoting the development of Tregs (53). The aberrant Warburg effect of tumor cells produces lactic acid to be exported into the extracellular space, which can result in an acidic TME. The resultant acidification of TME can induce the apoptosis of T cells and suppress T cell function by the inhibition of nuclear factor of activated T cells (NFAT) upregulation and the inhibition of p38 and JNK/c-Jun activation (54, 55). Lactic acid has also been shown to interfere with the maturation of DCs (56) and increase the frequency of forkhead box P3 (FoxP3)^+^ Tregs (57). Research has shown an increased expression of PD-L1 on tumor cells by the accumulation of lactic acid (58).

In addition to tumor cells, MDSCs and Tregs are two key cellular mediators in the immunosuppressive TME. The function of Tregs has been described above. Here, we discuss how MDSCs shape the intra-tumoral immune landscape to impair the function of T cells. MDSCs deplete amino acids in the TME that are essential for T cell function. MDSCs are characterized by the expression of enzyme arginase 1 (Arg1) (59). L-arginine is the substrate of Arg1, and excessive Arg1 leads to the depletion of L-arginine in the TME, which is of significant importance for the maturation of T cell receptor ζ-chain (TCRζ) and can therefore result in impaired T cell growth and differentiation (59). Moreover, MDSCs can deplete the extracellular cysteine pool to limit the activation of T cells (60). Inducible nitric oxide synthase-2 (iNOS2) produced by MDSCs can release high level of nitrogen monoxide (NO), which can interrupt T cell function by interfering with T cell JAK/STAT signaling proteins and can induce T cell apoptosis (61). MDSCs can also induce the proliferation of Tregs by secreting soluble factor IL-10 to further downregulate the activation and expansion of T cells (62).

Tumor vaccines can potentially induce efficient antitumor immunity by recruiting and activating immune cells. However, the mechanisms demonstrated above can be utilized by tumors to turn effector T cells into exhausted ones, which can be characterized by the deficiency of response to TCR stimulation, the production of cytokines and the upregulation of inhibitory factors. This condition can be associated with the loss of tumor growth control. In the following section, we consider how different strategies can modulate tumor vaccine function to better boost antitumor immunity.

## Strategies to Improve Tumor Vaccine Efficacy

Currently, strategies have been used in pre-clinical experiments and clinical trials to improve the anti-tumor efficacy of tumor vaccines. Here, we discuss how these strategies influence the responsiveness of tumor vaccines against tumors. **Table 1** shows the related strategies.

**Table 1:** Strategies enhancing the anti-tumor efficacy of tumor vaccines.

Strategies	Target steps^a^	Effect on tumor vaccines	Challenges
**1. Improve the design of tumor vaccines.**			
**Vaccines based on both MHC-I specific tumor antigens and MHC-II specific tumor antigens**	Inhibition of T cell priming and activation	CD4^+^ T cells help activate CD8^+^ T cells and help them mature into CTLs; more efficient uptake and presentation; cytokines secreted by CD4^+^ T cells dictate the quality of CTLs.	Loss of MHC-II molecules expression on tumor cells; few of found MHC-II restricted tumor antigens due to current prediction algorithms
**Re-assembling tumor vaccines with nanoparticles**	Inhibition of T cell priming and activation; inhibition of trafficking and infiltration to tumor bed; impairment of T cell function and the induction of dysfunctional T cells	Vectors for different molecules that synergize with tumor vaccines; stabilize the loaded adjuvants; reshape immunosuppressive TME; promote the production of CTLs.	The safety of chosen material compositions; optimize the physical properties of nanoparticles.
**2. Combine tumor vaccines with other therapies.**			
**Tumor vaccines plus metabolic therapy**	Impairment of T cell function and the induction of dysfunctional T cells	Switch to metabolisms beneficial to T cell function; magnify the metabolic plasticity defect of tumor cells.	Side effects caused by the dysfunctional normal cell metabolisms; intrinsic toxicity.
**Tumor vaccines plus CAR-T therapy**	inhibition of tumor antigen recognition	Recognize antigen on any HLA background; target tumor cells that downregulate MHC-I molecules.	Increased toxicity.
**Tumor vaccines plus ICBs therapy**	Inhibition of T cell priming and activation; impairment of T cell function and induction of dysfunctional T cells	Reinvigorate exhausted T cells; evoke tumor immunogenicity.	Induction of dysfunctional cell subsets; injection sequence.
**Tumor vaccines plus OVs therapy**	Inhibition of tumor antigen recognition; inhibition of T cell priming and activation; inhibition of trafficking and infiltration to tumor bed	Help reverse the down-regulation of MHC-I molecules; induce the maturation and function of DCs; reshape the immunosuppressive TME.	Balancing the antitumor immune response and antiviral immune response; more appropriate clinical indicators.
**Tumor vaccines plus epigenetic therapy**	Inhibition of tumor antigen recognition; inhibition of T cell priming and activation; inhibition of trafficking and infiltration to tumor bed; impairment of T cell function and the induction of dysfunctional T cells	Help restore APM expression; increase immune infiltration; directly reinvigorate exhausted T cells.	Intrinsic toxicity; balancing the pro-immunogenic and immunosuppressive functions.

^a^According to Modulation of T Cell Function by Tumors section, how tumors influence the function of T cells can be briefly concluded into the following steps: inhibition of tumor antigen recognition, inhibition of T cell priming and activation, inhibition of trafficking and infiltration to tumor bed, impairment of T cell function and the induction of dysfunctional T cells. This list mainly focuses on which steps the strategy targets to improve the function of tumor vaccines. MHC, major histocompatibility class; CTLs, cytotoxic T cells; TME, tumor microenvironment; ICBs, immune checkpoint blockage; APM, antigen processing machinery.

## Improve the Design of Current Tumor Vaccines

### Vaccines Based on Both MHC-I–Specific Tumor Antigens and MHC-II Specific Tumor Antigens

At present, most tumor immunotherapy is aimed at activating CD8^+^ T cells. With improved understanding of the immunology, recent studies have highlighted the role of CD4^+^ T cells in tumor clearance. The activation of an effective anti-tumor immune response not only needs helper T cells to recruit the killer T cells but also needs them induce killer T cells into the activation state in which they are capable of killing tumor cells (63, 64). In other words, killer T cells should synergize with the helper T cells to activate a robust and durable immune protection.

Studying mouse models of human tumors, researchers found that vaccines act more effectively when both helper and killer T cells are activated. Using the KP9025 sarcoma mice model, researchers indicated that the administration of vaccines with a mixture of KP.mLAMA4 (MHC-I specific tumor antigen) and KP.mITGB1 (MHC-II specific tumor antigen) provided more effective protection against tumor challenge than administration of each the antigen alone. What’s more, spleen from mice vaccinated with the mixture antigens contained more functional CD8^+^ T cells than the mice receiving only MHC-I specific tumor antigen. These results demonstrated the crucial role of CD4^+^ T cell in activating CD8^+^ T cells and helping them mature into CTLs (65), which may be the results of more efficient uptake and presentation by APCs (66, 67). In another study using the B16F10 tumor-bearing mice models, MHC class II-restricted neo-epitope-encoding RNA vaccines were injected into the mice. Compared with the control group, MDSCs and FoxP3^+^ Tregs were markedly reduced in the experimental group, indicating that the induction of CD4^+^ T cells could help overcome tumor-associated immune suppression, which in turn resulted in a more efficacious tumor control (68).

In fact, CD4^+^ T cells can indirectly promote anti-tumor responses, as the cytokines and co-stimulatory factors secreted by CD4^+^ T cells largely dictate the quality of CTLs. For example, IL-2 secreted by Th1 is essential for maintaining the growth and proliferation of CD8^+^ T cells (69). Moreover, Th1 can help promote the recruitment and infiltration of CD8^+^ T cells through the secretion of IFN-γ (70). Furthermore, tumor-specific CD4^+^ T cells can also secrete IL-4 to recruit macrophages as well as establish long-term memory immune responses to tumors (71, 72). In fact, immunotherapy based on mutation-specific CD4^+^ T cells has been proven effective in clinical trials. In patient 3737 experiencing widely metastatic cholangiocarcinoma, tumor infiltrating lymphocytes (TILs) containing approximately 25% mutation-specific Th1 cells were transferred, and the patient received tumor regression and experienced disease stabilization (NCT01174121) (73). *Patrick A. Ott* enrolled ten patients with previously untreated high-risk melanoma in a phase I study. In the study, they used long peptides leading to the activation of both CD8^+^ and CD4^+^ T cells. Of six patients treated with the vaccines, four had no recurrence at the follow up of 25 months after vaccination, while the two patients experiencing recurrent disease subsequently received anti-PD-1 therapy and achieved complete tumor regression (NCT 01970358) (8).

In conclusion, CD4^+^ T cells can help promote the effector function of CTLs and reshape the TME to overcome negative regulation, both of which can amplify the anti-tumor response of T cells. The reasons why tumor vaccines targeting CD4^+^ T cells are difficult to synthesize can be summarized into the following aspects. First, most tumors lack the expression of MHC-II molecules (68). Another factor may be the specificity of neoantigens in tumor cells. At present, most tumor neoantigens capable of stimulating effector T cells belong to MHC-I restricted molecules, and they can only be recognized by effector T cells. In contrast, few MHC-II restricted tumor neoantigens have been found. One of the obstacles is that the existing MHC-II antigen prediction algorithms find it difficult to identify MHC-II restricted tumor-specific antigens that function as neoantigens for CD4^+^ T cells (74).

Thinking of the importance of CD4^+^ T cells in antitumor immunity, it is urgent for us to deepen the understanding of the underlying principles of immunology and overcome technical difficulties before applying such vaccines into clinical practice.

### Re-Assembling Tumor Vaccines With Nanoparticles to Enhance the Immunogenicity

Due to the intricate immunosuppressive TME, the therapeutic effects of a majority of tumor vaccines are quite limited. The immunosuppressive TME inhibits not only the antigen uptake and presentation but also the activation and infiltration of lymphocytes *in vivo*. Therefore, new methods are expected to improve the effectiveness of current immunotherapies, and thus, nanoparticles have been in extensive use in recent years. Compared to traditional tumor vaccines, re-assembled tumor vaccines are armed with the following advantages brought by nano-materials.

First, nanoparticles can be loaded with different adjuvants and molecules, making them novel vectors for different types of tumor vaccines, such as peptide vaccines, RNA vaccines (75, 76). Molecules such as chemical drugs and immune checkpoint inhibitors can be loaded into the vectors with the vaccines to achieve synergistic antitumor effects. Liu X. et al. constructed a tumor nano-vaccine composed of antigenic peptide, CpG-ODN, and cationic polymer nanoparticle. Using breast carcinoma 4T1 models, they found that the injection of the nano-vaccine significantly promoted the infiltration of CTLs in the tumor and could help prevent tumor recurrence and pulmonary metastasis (77). Xie X. et al. developed a novel therapeutic vaccine co-encapsulating epitope peptide and PD-1 antibody with self-healing microcapsule. With the synergism of epitope peptide, PD-1 antibody, and the unique self-healing feature of the microcapsule, a single dose of the vaccine led to the recruitment of activated APCs and significantly improved the infiltration of tumor-specific CTLs. The results indicated that such new vaccine platform could serve as a promising immunotherapy modality for anti-tumor treatment (78).

Second, nanoparticles serve as safeguards to protect the adjuvants from being degraded in the biological environment and precisely deliver the therapeutic ingredients to a particular place to improve the immune response (79). Some particles may also release the coated ingredients according to the conditions of the environment, such as pH and oxygen content, to better adapt to the harsh TME. *Keman Cheng* developed a therapeutic peptide assembling nanoparticle that can sequentially respond to the tumor microenvironment. The basic of the nano-vaccine was a short D-peptide antagonist of programmed cell death-ligand 1 (^D^PPA-1), an inhibitor of idoleamine 2,3-dioxygenase (NLG919). When exposed to the acidic tumor microenvironment, the material became swollen. The material would soon collapse due to the cleavage of the peptide substrate by matrix metalloproteinase 2 (MMP-2), which is upregulated in tumor stroma. Such a subtle design guarantees the controllable release of the ingredient and provides a favorable circumstance for CTLs to survival and become activated (80).

Third, nano-materials can serve as an immunotherapeutic platform that can reshape immunosuppressive TME (81, 82). The low response rate of current tumor immunotherapy can partly account for the immunosuppressive factors in TME. To better overcome this, *Chanyoung Song* developed an injectable immunotherapeutic nanogel. The nanogel was injected in the dissected empty space after removing approximately 90% of the primary tumor by surgery in 4T1 triple-negative breast cancer and TC1 cervical cancer mice models. Compared with the groups without injected the nanogel, the original immunosuppressive TME of the experimental group was reverted into the immunogenic one, including increased levels of infiltrating CTLs and upregulated expression of cytokines. Moreover, the percentage of immunosuppressive cells such as MDSCs and M2 macrophages decreased after treatment (83).

Lastly, because the size of the nanoparticles is similar to that of the pathogens, the injection of the nanoparticles can promote the presentation of antigens, which can in turn induce the production of more effective, long-lasting tumor specific CTLs (84). Altogether, the modified tumor vaccines can effectively activate the host immune response and induce the death of tumor cells. The re-assembled tumor vaccines distributed in tumors reprogram the immunosuppressive networks and increase immune cells infiltration.

In spite of the fact that nanoparticles have enhanced the anti-tumor immune response in preclinical models, there remains difficulties before fully applying them to clinical use. One of the most important factors may be the selection of material compositions. The safety of the materials should achieve the certifications of the FDA. The materials should be biodegradable and their degradants must also be non-toxic to humans without leading to biological immune rejection. In addition to safety, the physical properties of these materials such as cation density and surface activity, need to be further optimized to better enhance the antitumor effect of the immune system.

## The Combination of Tumor Vaccines and Other Therapies

### The Combination of Tumor Vaccines and Metabolic Therapy

Both tumor cells and T cells are in desperate need of anabolic and energetic to maintain their survival and growth. Tumor cells are known to have unique metabolisms, and differences in cellular metabolism between tumor cells and immune cells can serve as a basis to better improve the antitumor effect.

The unique Warburg effect of tumor cells causes hypoglycemia and hypoxia in the TME (44). TILs must adapt to this environment to survive and exert anti-tumor function. By analyzing the CD8^+^ T cell metabolism 30 days after vaccine treatment in melanoma-bearing mice, Zhang et al. found that the intensity of glycolysis metabolites declined in CD8^+^ TILs while the peroxisome proliferator-activated receptor (PPAR)-a signaling and fatty acid (FA) catabolism metabolites increased, implying that CD8^+^ TILs switched to these modes of metabolism to preserve their effector functions under the pressure of hypoglycemia and hypoxia in TME. They further confirmed this conclusion by adding fenofibrate, a PPAR-a agonist, to increase FA catabolism. Indeed, the addition of fenofibrate largely improved the antitumor function of vaccines compared to the vaccinated, tumor-bearing group (85). Glutamine has long been used for metabolism therapy due to its crucial role in cell metabolism (86, 87). 6-diazo-5-oxo-L-norleucine (DON) is the most widely studied broad-spectrum glutamine antagonist and has been used in clinical trials (88, 89). However, DON was then abandoned as an antitumor agent due to its toxicity, especially gastrointestinal symptoms (88, 90). *Robert D. Leone* then developed a prodrug of DON, JHU083, which could only be activated in the TME. Blocking of glutamine metabolism led to the inhibition of wide-ranging metabolism and the disruption of NADP(H) homeostasis while robustly enhancing the function and effectiveness of TILs and acquiring a long-lived, highly activated phenotype. This difference may be due to the lack of plasticity in tumor metabolism (91).

In conclusion, metabolism is integrated in cellular processes and determines the fate of both tumor cells or immune cells. Deeper insights into the mechanisms of the differences between tumor cell and immune cell metabolism will help yield new targets for therapy.

### The Combination of Tumor Vaccines and Chemeric Antigen Receptor T Cells Therapy

CAR-T therapy is another successful attempt at immunotherapy. At present, dramatic clinical responses have been achieved in non-Hodgkin’s lymphoma (NHL), adult and pediatric patients with relapsed and refractory (R/R), B-cell acute lymphoblastic leukemia (B-ALL), and chronic lymphocytic leukemia (CLL) (92–94). However, relapse has become one of the obstacles that hinders the development of such therapy. It is well known that engraftment and persistence of CAR-T *in vivo* are the keys to successful tumor eradication. However, the frequency of CAR-T cells declined *in vivo* after transfer (95). The combination of tumor vaccine and CAR-T therapy will be a leap forward for tumor immunotherapy, as the combination can complement the shortcomings of each therapy alone and synergistically enhance anti-tumor ability. For example, unlike effector T cells activated by tumor vaccines which engage HLA-peptide complexes, CAR-T cells have the ability to recognize antigen on any HLA background and are therefore more broadly applicable to patient populations with diverse HLA background (96). They can also target tumor cells that downregulate MHC-I molecules, which is a major mechanism that contributes to tumor escape from vaccine therapy (97). Likewise, the administration of tumor vaccine improves the engraftment and persistence of CAR-T cells to partly overcome CAR-T cell exhaustion in the TME. Actually, a large number of studies have proved that this combination strategy is feasible.

*K. Reinhard* recently reported claudin (CLDN) 6 as a target for CAR-T cell therapy. In the study, the researchers designed 2^nd^ generation CLDN6-CAR engineered human T cells as well as a liposomal CLDN6-encoding RNA vaccine. Their data established that the addition of tumor vaccine induced a profound expansion of circulation CAR-T cells and showed complete rejection of tumors with higher median survival. The administration of tumor vaccine enabled tumor control at lower CAR-T cell doses (98).Similar results have been shown in other studies using WT1-specific CAR-T cells combined with DC vaccine pulsed with WT1_236Y_ peptide (99) and CMV/CD19 bispecific T cells combined with CMV peptide vaccine (100). In each study, the post-transfer of vaccine enhanced the therapeutic efficacy of CAR-T cells targeting tumor antigens *via* CAR-T cell expansion and activation. An important goal of immunotherapy is to establish immune memory to protect against tumor recurrence. In addition to the enhancement in anti-tumor ability, vaccines have been shown to increase the numbers of memory T cells and decrease the number of terminally differentiated T cells, implying that vaccines can promote immunologic memory in CAR-T cells (101). *Clare* rechallenged the long-term surviving mice with tumor cells in the contralateral site 100 days after the initial treatment, and they found that the mice were completely resistant to rechallenge with the same tumor cells, suggesting that the administration of vaccine did generate a memory response in the surviving mice (102).

The concept of the combination of tumor vaccine and CAR-T cell therapy to specifically eradicate malignant cells has been repeatedly demonstrated in animal models but was sometimes questioned due to the results of trials in humans. In a phase 1/2 study of WT1_236Y_ peptide vaccine involving 26 patients, a patient suffered from an immune-related, but manageable, adverse event (103). Therefore, there remains the possibility that the synergism of tumor vaccine and CAR-T therapy may be associated with increased toxicity. The combination of vaccine and CAR-T therapy has been applied in a relapsed pediatric acute lymphoblastic leukemia clinical trial (NCT01195480). In the CD19CAR CTL therapy with irradiated EBV transformed lymphoblastoid cell lines (LCL) vaccination cohort, four of six patients had detectable CAR-T cells in the blood until 1–3 months after infusion and showed a significantly improved persistence compared with the cohort without vaccination. However, while the usage of vaccine improved the persistence of CAR-T cells somewhat, it was inadequate to induce the proliferation of CAR19 CTLs needed for an effective antitumor response (104). Such results require us to take how to mitigate such risks into more careful consideration and to refine the combination therapeutic strategies, including the doses of tumor vaccine and CAR-T cells in combination therapy, the injection time and the injection sequence.

### The Combination of Tumor Vaccines and Immune Checkpoint Blockade Immunotherapy

Growing preclinical and clinical trials have combined tumor vaccines with ICBs in the attempt to reinvigorate exhausted T cells, and many support the assumption that the combination of ICBs and tumor vaccines have the ability to synergistically improve the clinical outcome (105–108). The immune checkpoint pathway plays an important role in maintaining immune tolerance and deciding the fate and function of T cells (109). Herein is discussed the optimal timing and sequencing of the combination of ICBs and tumor vaccines to obtain the maximum therapeutic benefits.

Because the preparation of tumor vaccines will take a long period of time, ICBs have always been the priority selection in the clinic, followed by tumor vaccines. However, studies have shown that the initial state of T cells is one of the main reasons affecting the effect of combination treatment. Vivek Verma et al. (110) uncovered the phenomenon that when PD-1 was blocked first, the anti-tumor effect of the tumor vaccines was abrogated due to the decrease rate of CD8^+^ T cells and defective T cell activation. The blockade of PD-1 under sub-optimally activated CD8^+^ T cell conditions would lead to the presence of dysfunctional PD-1^+^CD38^hi^CD8^+^ T cells, resulting in the failure of antigenic stimulation response and defective effector functions (111). Researchers have found that such populations of T cells are associated with resistance to anti-PD-1 therapy. If treated appropriately with tumor vaccines before PD-1 inhibitors, the resistance to the PD-1 inhibitors will be resolved. This finding implies that proper sequencing of immunomodulatory agents is necessary for ideal clinical outcomes. In a therapeutic strategy for the clinical use of PD-1 inhibitors combined with tumor vaccines, it may be better to use tumor vaccines before PD-1 inhibitors, or at least use them at the same time. However, we come to a totally opposite conclusion in another experiment using ARF-Fc anti-CTLA-4 mAb where if anti-CTLA-4 mAb was given with the peptide vaccines simultaneously, not only the CTLA-4^+^ Tregs but also the CTLA-4^+^CD8^+^ T cells were depleted. Giving the anti-CTLA-4 mAb treatment several days before the vaccine stimulation resulted in the depletion of Tregs and the expansion of antigen-specific CD8^+^ T cells *in vitro* and led to an enhanced anti-tumor effect *in vivo* (112). Whether all the ICBs would undergo the above changes when combined with the tumor vaccines needs to be further confirmed. However, these reports do provide unique insights into the normalization of the combined immunomodulatory agents.

In conclusion, correct timing and sequence of ICBs treatment and tumor antigen vaccines are important factors influencing the effect of tumor immunotherapy. Such normalization should be based on the kinetics of immune checkpoints and effector T cell activation.

### The Combination of Tumor Vaccines and Oncolytic Viruses

To better improve the antitumor efficacy of tumor vaccines, the ability to alter the immunosuppressive TME is the most attractive feature. When compared with other immunotherapies, OVs seem to be more ideal antitumor immunity inducers. OVs have been shown to overcome the issues regarding immunosuppressive TME, which often encountered by T cell therapies.

OVs can promote antitumor T cell responses through multiple mechanisms. First, OVs can promote T cell priming. Due to the defective antiviral responses, tumor cells are susceptible to virus infection. The enzymes and factors required for rapid cell division are often highly expressed in tumor cells, providing viruses with a replicative advantage in the tumor cells (1). Once the infection is established, continuous replication of viruses will at last lead to oncolysis. When the tumor cells are broken, the damage associated molecular patterns and tumor antigens will be released into the TME, which can contribute to the maturation and function of DCs, leading to the priming of tumor-specific T cell response (113, 114). Second, OVs induce a pro-inflammatory TME that can increase T cell trafficking and infiltration. Chemokines and cytokines regulate the trafficking and infiltration of immune cells into the TME. Studies have shown that the infection of OVs elicits potent type I interferon responses (115, 116), which can stimulate the production of T cell recruiting chemokines. Inflammatory factors such as tumor necrosis factor (TNF) and interleukin-1β (IL-1β) can also be induced by OVs, which in turn upregulate the expression of selectin on endothelial cells and provide an important signal for T cell infiltration (117–119). The presence of physical barriers is another critical reason accounting for the defective T cell infiltration. However, Ilkow et al. found that the vesicular stomatitis virus (VSV) can selectively destroy cancer-associated fibroblasts (CAFs), which are key components of immunosuppressive tumor stroma (120). Their depletion can represent yet another means by which OVs increase the infiltration of T cells. Last, OVs improve the recognition of tumor cells. To avoid T cell recognition, tumor cells can downregulate MHC class I expression and other components involved in the antigen processing and presentation pathway. OVs have the ability to reverse these effects, likely by inducing type I interferon production (121, 122).

Bringing together the above concepts, combined treatment with OVs can be a promising strategy for reverting immunosuppressive TME and enhancing the antitumor capabilities of tumor vaccines. In B16-OVA melanoma mouse models, combination treatment of VSV-GP, a chimeric vesicular stomatitis virus (VSV) pseudotyped with the glycoprotein (GP) of the lymphocytic choriomeningitis virus, with an ovalbumin (OVA) peptide-loaded dendritic cell (DC) vaccine (DCVacc) significantly increased the numbers of tumor-infiltrating, highly activated T cells while relatively reducing the number of Tregs. Researchers observed that several proinflammatory cytokines increased in the VSV-GP-treated group (123). Using a pre-clinical ovarian cancer mouse model, the combination of vaccine and antigen-armed oncolytic Maraba virus elicited robust tumor-specific CD8^+^ T cell responses and led to unique immunological changes that correlated with improved clinical outcome of ovarian cancer patients (124). These findings pointed to a key role of OVs to help exert systemic immunologic effects and improve survival. OVs can also serve as adjuvants for tumor vaccines. Erkko Ylösmäki et al. physically attached tumor-specific peptides onto the viral envelope of the virus and found that by coating the viral envelope with therapeutic peptides, the antitumor immunity in the tumor microenvironment can be significantly enhanced (125).

In conclusion, the synergism of tumor vaccine and OVs can combine the advantages of both approaches. The viruses activate the immune response and reshape the TME, and the therapeutic antitumor vaccine direct the immune response towards the neoantigens. Currently, numerous oncolytic viruses have been applied in early phase clinical trials (126–128). Unlike standard drugs, such live viruses have unique challenges. They are live viruses and can proliferate during clinical administration, making it difficult to establish safe and effective dosing guidelines. Moreover, the immune response induced by OVs can be further divided into antitumor immune response and antiviral immune response. On the one hand, viruses help induce immune response against tumor cells. On the other hand, the response to neutralize virus toxicity may block virus replication and the ongoing infection of tumor cells (129). Hence, more careful attention should be taken in the establishment of this combination therapeutic regimen to balance the immune responses and maximize the advantages of the combination therapy. Moreover, compared to agents that directly kill tumor cells, immune-mediated antitumor responses will be much slower. Therefore, more appropriate clinical indicators are needed to capture therapeutic responses of the combination therapy.

### The Combination of Tumor Vaccines and Epigenetic Therapy

Immune cells infiltrating in the TME are frequently in an exhaustion state and accumulating evidence indicates that such reprogramming can partly be the consequence of aberrant epigenomes. As a result, epigenetic therapy has the potential to reverse the exhausted immune T cells caused by aberrant epigenomes. At present, histone deacetylase inhibitors (HDACi) and hypomethylating agents have been approved by the FDA and have brought epigenetic therapy to the front of tumor therapies. Collectively, the synergistic effect of epigenetic drugs on tumor vaccines can be concluded in the following points.

First, epigenetic therapy helps restore the HLA class-I antigen processing machinery expression on tumors. For example, hypomethylating agents have been shown to have the ability to upregulate tumor antigens as well as antigen processing and presentation genes (130, 131). Similarly, in the glioma implantation mouse model, Ting Sun et al. found upregulated expression of antigen processing and presenting associated molecules on the surface of the glioma tumor cells in the HDACi-treated group, such as TAP1, TAP2, and MHC-I, thereby enhancing the specific lysing efficacy of the immune cells and in turn potentiating the immune response (132). Interestingly, Ailsa et al. (133) uncovered that HDACi mediated tumor cells apoptosis could stimulate the uptake by APCs and that the combination of HDACi and immune-activating antibodies to promote the function of APCs could enhance the proliferation and survival of cytotoxic T cells. Second, epigenetic therapy can modulate the immunosuppressive TME. Both hypomethylating agents and HDACi have been shown to increase the number of natural killer (NK) cells in the tumor bed while reducing the percentage of tumor-infiltrating MDSCs and Tregs (134–136). Tumor cells can epigenetically silence the expression of chemokines to impair the infiltration activity of immune cells. However, pharmacological inhibition of histone deacetylase in tumor cells was shown to increase the expression of chemokines, thus attracting peripheral infiltrating T cells to the tumor bed (27). Last, apart from regulating T cell extrinsic factors, epigenetic therapy can act on TILs directly by re-invigorating exhausted T cells (137) and decreasing the activation induced cell death in TILs (138). However, it is worth mentioning that epigenetic therapy can also suppress the immune response. For example, the administration of hypomethylating agents prior to allogeneic transplantation can relieve graft versus-host disease (GVHD) through inducing the production of Tregs (139), which implies that it is necessary to balance the pro-immunogenic and immunosuppressive functions in clinical use.

Generally, clinical agents that can be used in conjunction with tumor vaccines should have the characteristics below. First, they act directly upon the T cells to enhance the adaptive anti-tumor immune response. Second, they relieve the tumor-induced immunosuppression, which in turn provides a suitable immune microenvironment for anti-tumor immune cells to help them work properly. Considering the fact that some drugs may also produce cytotoxic effect on immune cells, it is critical to ensure that appropriate dosages are used in the combination therapy.

## Discussion

In the TME, T cells are key cellular components that extensively crosstalk with tumor cells. Recent successes have fueled interest in improving the durable anti-tumor ability of tumor vaccines. Advances in the understanding of immune regulation mechanisms provide solid foundation for the development of novel tumor vaccine combination therapy strategies. However, the occurrence and development of tumors is a dynamic evolution process characterized by genetic instability (1). Due to the immune escape mechanism of tumor cells, tumor vaccines alone may not exert expected tumor killing effects, which is one of the factors that hinders the application of tumor vaccines. In the upcoming era, the application of tumor vaccines should be combined with other therapies such as chemotherapy, radiation therapy, molecular targeted drugs and other immunotherapies to produce a more durable anti-tumor immune response and improve the prognosis of cancer patients.

Although combined treatments have achieved anti-tumor effects and prolonged survival time in pre-clinical animal models, questions exist in clinical translation. The most important point is that the immune background of the animal models is quite different from that of humans. Although patient-derived tumor xenograft (PDX) models have been widely applied in tumor immunology research, the dosage and usage in animal models still require a long time to verify their feasibility in human beings, which greatly extends the time for clinical translation. Second, due to the complexity of tumor immunotherapy and the heterogeneity of patients in the clinic, more precise predictive biomarkers still need to be explored to better identify the standard to combine for different treatments. They can also help to determine which treatments are suitable for patients in early clinical diagnosis to develop personal treatment options for different patients. The occurrence and development of tumors is a complex process. Under the pressure of immune survival, a series of changes will happen in tumor cells to avoid immune responses. These lead to challenges in clinical work of how to adjust the combined treatment regimen in time according to different biological changes in tumor cells to maximize the effect of combined treatment. Third, like predictive biomarkers, appropriate indicators should also be developed in the clinic to assess the effect of combination therapy. Finally, a perfect combination treatment regimen needs to be developed based on the cancer immunity cycle to decide which treatment should be included. In other words, such regimens should include activating the endogenous immune response, promoting immune cell infiltration, increasing tumor sensitivity to therapy, reducing tumor burden and maintaining long-term immunity. This requires clinicians to have an accurate grasp of the combination treatment regimen. Continuous studies on tumor immune mechanism and clinical translation are needed to overcome the questions of how to combine different treatments; whether each of the treatment should be added sequentially or concurrently; and whether each of the treatments should be added continuously of intermittently. In general, the biological toxicity of each treatment should be minimized while the synergistic effects of each treatment should be maximized.

## Author Contributions

JC, HZ, LZ, and YHu reviewed relevant literatures and drafted the manuscript. ML, YHe, and YL analyzed and revised the manuscript. All authors contributed to the article and approved the submitted version.

## Funding

This work was funded by the Frontier Research Program of Bioland Laboratory (Guangzhou Regenerative Medicine and Health Guangdong Laboratory) [grant number 2018GZR110105014], the Natural Science Foundation of Guangdong Province, China [grant number 2018B030311042], Science and Technology Program of Guangzhou, China [grant number 201704020216], Clinical Research Startup Program of Southern Medical University by High-level University Construction Funding of Guangdong Provincial Department of Education [grant number LC2016ZD027], and President Foundation of Zhujiang Hospital, Southern Medical University [grant number yjjj2019qn07].

## Conflict of Interest

The authors declare that the research was conducted in the absence of any commercial or financial relationships that could be construed as a potential conflict of interest.

## References

[1] Hanahan D Weinberg RA Hallmarks of cancer: the next generation. Cell (2011) 144(5):646–74. 10.1016/j.cell.2011.02.013 21376230

[2] De Plaen E Lurquin C Van Pel A Mariame B Szikora JP Wolfel T Immunogenic (tum-) variants of mouse tumor P815: cloning of the gene of tum- antigen P91A and identification of the tum- mutation. Proc Natl Acad Sci U S A (1988) 85(7):2274–8. 10.1073/pnas.85.7.2274 PMC279973 3127830

[3] Boon T Cerottini JC Van den Eynde B van der Bruggen P Van Pel A Tumor antigens recognized by T lymphocytes. Annu Rev Immunol (1994) 12:337–65. 10.1146/annurev.iy.12.040194.002005 8011285

[4] Xing Y Hogquist KA T-cell tolerance: central and peripheral. Cold Spring Harb Perspect Biol (2012) 4(6):a006957. 10.1101/cshperspect.a006957 22661634 PMC3367546

[5] Aleksic M Liddy N Molloy PE Pumphrey N Vuidepot A Chang KM Different affinity windows for virus and cancer-specific T-cell receptors: implications for therapeutic strategies. Eur J Immunol (2012) 42(12):3174–9. 10.1002/eji.201242606 PMC3776049 22949370

[6] van Buuren MM Calis JJ Schumacher TN High sensitivity of cancer exome-based CD8 T cell neo-antigen identification. Oncoimmunology (2014) 3:e28836. 10.4161/onci.28836 25083320 PMC4106163

[7] Yadav M Jhunjhunwala S Phung QT Lupardus P Tanguay J Bumbaca S Predicting immunogenic tumour mutations by combining mass spectrometry and exome sequencing. Nature (2014) 515(7528):572–6. 10.1038/nature14001 25428506

[8] Ott PA Hu Z Keskin DB Shukla SA Sun J Bozym DJ An immunogenic personal neoantigen vaccine for patients with melanoma. Nature (2017) 547(7662):217–21. 10.1038/nature22991 PMC5577644 28678778

[9] Sahin U Derhovanessian E Miller M Kloke BP Simon P Lower M Personalized RNA mutanome vaccines mobilize poly-specific therapeutic immunity against cancer. Nature (2017) 547(7662):222–6. 10.1038/nature23003 28678784

[10] Chen DS Mellman I Oncology meets immunology: the cancer-immunity cycle. Immunity (2013) 39(1):1–10. 10.1016/j.immuni.2013.07.012 23890059

[11] Seliger B Molecular mechanisms of MHC class I abnormalities and APM components in human tumors. Cancer Immunol Immunother (2008) 57(11):1719–26. 10.1007/s00262-008-0515-4 PMC11030176 18408926

[12] Garrido F Algarra I Garcia-Lora AM The escape of cancer from T lymphocytes: immunoselection of MHC class I loss variants harboring structural-irreversible “hard” lesions. Cancer Immunol Immunother (2010) 59(10):1601–6. 10.1007/s00262-010-0893-2 PMC11029827 20625726

[13] Garcia-Lora A Algarra I Garrido F MHC class I antigens, immune surveillance, and tumor immune escape. J Cell Physiol (2003) 195(3):346–55. 10.1002/jcp.10290 12704644

[14] Abele R Tampe R Modulation of the antigen transport machinery TAP by friends and enemies. FEBS Lett (2006) 580(4):1156–63. 10.1016/j.febslet.2005.11.048 16359665

[15] Einstein MH Leanza S Chiu LG Schlecht NF Goldberg GL Steinberg BM Genetic variants in TAP are associated with high-grade cervical neoplasia. Clin Cancer Res (2009) 15(3):1019–23. 10.1158/1078-0432.CCR-08-1207 PMC5932631 19188174

[16] Leibowitz MS Andrade Filho PA Ferrone S Ferris RL Deficiency of activated STAT1 in head and neck cancer cells mediates TAP1-dependent escape from cytotoxic T lymphocytes. Cancer Immunol Immunother (2011) 60(4):525–35. 10.1007/s00262-010-0961-7 PMC3426276 21207025

[17] Marincola FM Jaffee EM Hicklin DJ Ferrone S Escape of human solid tumors from T-cell recognition: molecular mechanisms and functional significance. Adv Immunol (2000) 74:181–273. 10.1016/s0065-2776(08)60911-6 10605607

[18] Khan AN Gregorie CJ Tomasi TB Histone deacetylase inhibitors induce TAP, LMP, Tapasin genes and MHC class I antigen presentation by melanoma cells. Cancer Immunol Immunother (2008) 57(5):647–54. 10.1007/s00262-007-0402-4 PMC3146348 18046553

[19] Chattopadhyay G Shevach EM Antigen-specific induced T regulatory cells impair dendritic cell function via an IL-10/MARCH1-dependent mechanism. J Immunol (2013) 191(12):5875–84. 10.4049/jimmunol.1301693 PMC3858537 24218453

[20] Wing K Onishi Y Prieto-Martin P Yamaguchi T Miyara M Fehervari Z CTLA-4 control over Foxp3+ regulatory T cell function. Science (2008) 322(5899):271–5. 10.1126/science.1160062 18845758

[21] Akkaya B Oya Y Akkaya M Al Souz J Holstein AH Kamenyeva O Regulatory T cells mediate specific suppression by depleting peptide-MHC class II from dendritic cells. Nat Immunol (2019) 20(2):218–31. 10.1038/s41590-018-0280-2 PMC6402611 30643268

[22] Andrews LP Marciscano AE Drake CG Vignali DA LAG3 (CD223) as a cancer immunotherapy target. Immunol Rev (2017) 276(1):80–96. 10.1111/imr.12519 28258692 PMC5338468

[23] Marijt KA Sluijter M Blijleven L Tolmeijer SH Scheeren FA van der Burg SH Metabolic stress in cancer cells induces immune escape through a PI3K-dependent blockade of IFNgamma receptor signaling. J Immunother Cancer (2019) 7(1):152. 10.1186/s40425-019-0627-8 31196219 PMC6567539

[24] Chemnitz JM Parry RV Nichols KE June CH Riley JL SHP-1 and SHP-2 associate with immunoreceptor tyrosine-based switch motif of programmed death 1 upon primary human T cell stimulation, but only receptor ligation prevents T cell activation. J Immunol (2004) 173(2):945–54. 10.4049/jimmunol.173.2.945 15240681

[25] Bedognetti D Spivey TL Zhao Y Uccellini L Tomei S Dudley ME CXCR3/CCR5 pathways in metastatic melanoma patients treated with adoptive therapy and interleukin-2. Br J Cancer (2013) 109(9):2412–23. 10.1038/bjc.2013.557 PMC3817317 24129241

[26] Barreira da Silva R Laird ME Yatim N Fiette L Ingersoll MA Albert ML Dipeptidylpeptidase 4 inhibition enhances lymphocyte trafficking, improving both naturally occurring tumor immunity and immunotherapy. Nat Immunol (2015) 16(8):850–8. 10.1038/ni.3201 26075911

[27] Peng D Kryczek I Nagarsheth N Zhao L Wei S Wang W Epigenetic silencing of TH1-type chemokines shapes tumour immunity and immunotherapy. Nature (2015) 527(7577):249–53. 10.1038/nature15520 PMC4779053 26503055

[28] Dangaj D Bruand M Grimm AJ Ronet C Barras D Duttagupta PA Cooperation between Constitutive and Inducible Chemokines Enables T Cell Engraftment and Immune Attack in Solid Tumors. Cancer Cell (2019) 35(6):885–900.e10. 10.1016/j.ccell.2019.05.004 31185212 PMC6961655

[29] Spranger S Bao R Gajewski TF Melanoma-intrinsic beta-catenin signalling prevents anti-tumour immunity. Nature (2015) 523(7559):231–5. 10.1038/nature14404 25970248

[30] Fujihara T Yashiro M Inoue T Sawada T Kato Y Ohira M Decrease in ICAM-1 expression on gastric cancer cells is correlated with lymph node metastasis. Gastric Cancer (1999) 2(4):221–5. 10.1007/s101200050067 11957102

[31] Maeda K Kang SM Sawada T Nishiguchi Y Yashiro M Ogawa Y Expression of intercellular adhesion molecule-1 and prognosis in colorectal cancer. Oncol Rep (2002) 9(3):511–4. 11956618

[32] Piali L Fichtel A Terpe HJ Imhof BA Gisler RH Endothelial vascular cell adhesion molecule 1 expression is suppressed by melanoma and carcinoma. J Exp Med (1995) 181(2):811–6. 10.1084/jem.181.2.811 PMC2191895 7530765

[33] Afanasiev OK Nagase K Simonson W Vandeven N Blom A Koelle DM Vascular E-selectin expression correlates with CD8 lymphocyte infiltration and improved outcome in Merkel cell carcinoma. J Invest Dermatol (2013) 133(8):2065–73. 10.1038/jid.2013.36 PMC3644376 23353989

[34] Wu X Giobbie-Hurder A Liao X Lawrence D McDermott D Zhou J VEGF Neutralization Plus CTLA-4 Blockade Alters Soluble and Cellular Factors Associated with Enhancing Lymphocyte Infiltration and Humoral Recognition in Melanoma. Cancer Immunol Res (2016) 4(10):858–68. 10.1158/2326-6066.CIR-16-0084 PMC5050160 27549123

[35] Shin SJ Jeon YK Cho YM Lee JL Chung DH Park JY The Association Between PD-L1 Expression and the Clinical Outcomes to Vascular Endothelial Growth Factor-Targeted Therapy in Patients With Metastatic Clear Cell Renal Cell Carcinoma. Oncologist (2015) 20(11):1253–60. 10.1634/theoncologist.2015-0151 PMC4718425 26424759

[36] Seeber A Klinglmair G Fritz J Steinkohl F Zimmer KC Aigner F High IDO-1 expression in tumor endothelial cells is associated with response to immunotherapy in metastatic renal cell carcinoma. Cancer Sci (2018) 109(5):1583–91. 10.1111/cas.13560 PMC5980224 29498788

[37] Lim WC Olding M Healy E Millar TM Human Endothelial Cells Modulate CD4(+) T Cell Populations and Enhance Regulatory T Cell Suppressive Capacity. Front Immunol (2018) 9:565. 10.3389/fimmu.2018.00565 29628925 PMC5876242

[38] Nambiar DK Aguilera T Cao H Kwok S Kong C Bloomstein J Galectin-1-driven T cell exclusion in the tumor endothelium promotes immunotherapy resistance. J Clin Invest (2019) 129(12):5553–67. 10.1172/JCI129025 PMC6877340 31710313

[39] Kraan J van den Broek P Verhoef C Grunhagen DJ Taal W Gratama JW Endothelial CD276 (B7-H3) expression is increased in human malignancies and distinguishes between normal and tumour-derived circulating endothelial cells. Br J Cancer (2014) 111(1):149–56. 10.1038/bjc.2014.286 PMC4090744 24892449

[40] Kim CG Jang M Kim Y Leem G Kim KH Lee H VEGF-A drives TOX-dependent T cell exhaustion in anti-PD-1-resistant microsatellite stable colorectal cancers. Sci Immunol (2019) 4(41):eaay0555. 10.1126/sciimmunol.aay0555 31704735

[41] Sata M Walsh K TNFalpha regulation of Fas ligand expression on the vascular endothelium modulates leukocyte extravasation. Nat Med (1998) 4(4):415–20. 10.1038/nm0498-415 PMC2828686 9546786

[42] Jain RK Normalization of tumor vasculature: an emerging concept in antiangiogenic therapy. Science (2005) 307(5706):58–62. 10.1126/science.1104819 15637262

[43] Wigerup C Pahlman S Bexell D Therapeutic targeting of hypoxia and hypoxia-inducible factors in cancer. Pharmacol Ther (2016) 164:152–69. 10.1016/j.pharmthera.2016.04.009 27139518

[44] Warburg O On the origin of cancer cells. Science (1956) 123(3191):309–14. 10.1126/science.123.3191.309 13298683

[45] Chang CH Qiu J O’Sullivan D Buck MD Noguchi T Curtis JD Metabolic Competition in the Tumor Microenvironment Is a Driver of Cancer Progression. Cell (2015) 162(6):1229–41. 10.1016/j.cell.2015.08.016 PMC4864363 26321679

[46] Ho PC Bihuniak JD Macintyre AN Staron M Liu X Amezquita R Phosphoenolpyruvate Is a Metabolic Checkpoint of Anti-tumor T Cell Responses. Cell (2015) 162(6):1217–28. 10.1016/j.cell.2015.08.012 PMC4567953 26321681

[47] Munn DH Shafizadeh E Attwood JT Bondarev I Pashine A Mellor AL Inhibition of T cell proliferation by macrophage tryptophan catabolism. J Exp Med (1999) 189(9):1363–72. 10.1084/jem.189.9.1363 PMC2193062 10224276

[48] Fallarino F Grohmann U You S McGrath BC Cavener DR Vacca C The combined effects of tryptophan starvation and tryptophan catabolites down-regulate T cell receptor zeta-chain and induce a regulatory phenotype in naive T cells. J Immunol (2006) 176(11):6752–61. 10.4049/jimmunol.176.11.6752 16709834

[49] Pallett LJ Schmidt N Schurich A T cell metabolism in chronic viral infection. Clin Exp Immunol (2019) 197(2):143–52. 10.1111/cei.13308 PMC6642876 31038727

[50] Currie E Schulze A Zechner R Walther TC Farese RV Jr. Cellular fatty acid metabolism and cancer. Cell Metab (2013) 18(2):153–61. 10.1016/j.cmet.2013.05.017 PMC3742569 23791484

[51] Walton ZE Patel CH Brooks RC Yu Y Ibrahim-Hashim A Riddle M Acid Suspends the Circadian Clock in Hypoxia through Inhibition of mTOR. Cell (2018) 174(1):72–87.e32. 10.1016/j.cell.2018.05.009 29861175 PMC6398937

[52] Zheng Y Delgoffe GM Meyer CF Chan W Powell JD Anergic T cells are metabolically anergic. J Immunol (2009) 183(10):6095–101. 10.4049/jimmunol.0803510 PMC2884282 19841171

[53] Battaglia M Stabilini A Roncarolo MG Rapamycin selectively expands CD4+CD25+FoxP3+ regulatory T cells. Blood (2005) 105(12):4743–8. 10.1182/blood-2004-10-3932 15746082

[54] Mendler AN Hu B Prinz PU Kreutz M Gottfried E Noessner E Tumor lactic acidosis suppresses CTL function by inhibition of p38 and JNK/c-Jun activation. Int J Cancer (2012) 131(3):633–40. 10.1002/ijc.26410 21898391

[55] Brand A Singer K Koehl GE Kolitzus M Schoenhammer G Thiel A LDHA-Associated Lactic Acid Production Blunts Tumor Immunosurveillance by T and NK Cells. Cell Metab (2016) 24(5):657–71. 10.1016/j.cmet.2016.08.011 27641098

[56] Gottfried E Kunz-Schughart LA Ebner S Mueller-Klieser W Hoves S Andreesen R Tumor-derived lactic acid modulates dendritic cell activation and antigen expression. Blood (2006) 107(5):2013–21. 10.1182/blood-2005-05-1795 16278308

[57] Comito G Iscaro A Bacci M Morandi A Ippolito L Parri M Lactate modulates CD4(+) T-cell polarization and induces an immunosuppressive environment, which sustains prostate carcinoma progression via TLR8/miR21 axis. Oncogene (2019) 38(19):3681–95. 10.1038/s41388-019-0688-7 30664688

[58] Feng J Yang H Zhang Y Wei H Zhu Z Zhu B Tumor cell-derived lactate induces TAZ-dependent upregulation of PD-L1 through GPR81 in human lung cancer cells. Oncogene (2017) 36(42):5829–39. 10.1038/onc.2017.188 28604752

[59] Szefel J Danielak A Kruszewski WJ Metabolic pathways of L-arginine and therapeutic consequences in tumors. Adv Med Sci (2019) 64(1):104–10. 10.1016/j.advms.2018.08.018 30605863

[60] Srivastava MK Sinha P Clements VK Rodriguez P Ostrand-Rosenberg S Myeloid-derived suppressor cells inhibit T-cell activation by depleting cystine and cysteine. Cancer Res (2010) 70(1):68–77. 10.1158/0008-5472.CAN-09-2587 20028852 PMC2805057

[61] Waldron TJ Quatromoni JG Karakasheva TA Singhal S Rustgi AK Myeloid derived suppressor cells: Targets for therapy. Oncoimmunology (2013) 2(4):e24117. 10.4161/onci.24117 23734336 PMC3654606

[62] Serafini P Mgebroff S Noonan K Borrello I Myeloid-derived suppressor cells promote cross-tolerance in B-cell lymphoma by expanding regulatory T cells. Cancer Res (2008) 68(13):5439–49. 10.1158/0008-5472.CAN-07-6621 PMC2887390 18593947

[63] Zhu Z Cuss SM Singh V Gurusamy D Shoe JL Leighty R CD4+ T Cell Help Selectively Enhances High-Avidity Tumor Antigen-Specific CD8+ T Cells. J Immunol (2015) 195(7):3482–9. 10.4049/jimmunol.1401571 PMC7687044 26320256

[64] Borst J Ahrends T Babala N Melief CJM Kastenmuller W CD4(+) T cell help in cancer immunology and immunotherapy. Nat Rev Immunol (2018) 18(10):635–47. 10.1038/s41577-018-0044-0 30057419

[65] Alspach E Lussier DM Miceli AP Kizhvatov I DuPage M Luoma AM MHC-II neoantigens shape tumour immunity and response to immunotherapy. Nature (2019) 574(7780):696–701. 10.1038/s41586-019-1671-8 31645760 PMC6858572

[66] Bennett SR Carbone FR Karamalis F Miller JF Heath WR Induction of a CD8+ cytotoxic T lymphocyte response by cross-priming requires cognate CD4+ T cell help. J Exp Med (1997) 186(1):65–70. 10.1084/jem.186.1.65 9206998 PMC2198964

[67] Bennett SR Carbone FR Karamalis F Flavell RA Miller JF Heath WR Help for cytotoxic-T-cell responses is mediated by CD40 signalling. Nature (1998) 393(6684):478–80. 10.1038/30996 9624004

[68] Kreiter S Vormehr M van de Roemer N Diken M Lower M Diekmann J Mutant MHC class II epitopes drive therapeutic immune responses to cancer. Nature (2015) 520(7549):692–6. 10.1038/nature14426 PMC4838069 25901682

[69] Greenberg PD Adoptive T cell therapy of tumors: mechanisms operative in the recognition and elimination of tumor cells. Adv Immunol (1991) 49:281–355. 10.1016/s0065-2776(08)60778-6 1853786

[70] Bos R Sherman LA CD4+ T-cell help in the tumor milieu is required for recruitment and cytolytic function of CD8+ T lymphocytes. Cancer Res (2010) 70(21):8368–77. 10.1158/0008-5472.Can-10-1322 PMC2970736 20940398

[71] Quezada SA Simpson TR Peggs KS Merghoub T Vider J Fan X Tumor-reactive CD4(+) T cells develop cytotoxic activity and eradicate large established melanoma after transfer into lymphopenic hosts. J Exp Med (2010) 207(3):637–50. 10.1084/jem.20091918 PMC2839156 20156971

[72] Xie Y Akpinarli A Maris C Hipkiss EL Lane M Kwon EK Naive tumor-specific CD4(+) T cells differentiated in vivo eradicate established melanoma. J Exp Med (2010) 207(3):651–67. 10.1084/jem.20091921 PMC2839147 20156973

[73] Tran E Turcotte S Gros A Robbins PF Lu YC Dudley ME Cancer immunotherapy based on mutation-specific CD4+ T cells in a patient with epithelial cancer. Science (2014) 344(6184):641–5. 10.1126/science.1251102 PMC6686185 24812403

[74] Andreatta M Trolle T Yan Z Greenbaum JA Peters B Nielsen M An automated benchmarking platform for MHC class II binding prediction methods. Bioinformatics (2018) 34(9):1522–8. 10.1093/bioinformatics/btx820 PMC5925780 29281002

[75] Liu L Wang Y Miao L Liu Q Musetti S Li J Combination Immunotherapy of MUC1 mRNA Nano-vaccine and CTLA-4 Blockade Effectively Inhibits Growth of Triple Negative Breast Cancer. Mol Ther (2018) 26(1):45–55. 10.1016/j.ymthe.2017.10.020 29258739 PMC5763160

[76] Shan W Zheng H Fu G Liu C Li Z Ye Y Bioengineered Nanocage from HBc Protein for Combination Cancer Immunotherapy. Nano Lett (2019) 19(3):1719–27. 10.1021/acs.nanolett.8b04722 30724087

[77] Liu X Feng Z Wang C Su Q Song H Zhang C Co-localized delivery of nanomedicine and nanovaccine augments the postoperative cancer immunotherapy by amplifying T-cell responses. Biomaterials (2020) 230:119649. 10.1016/j.biomaterials.2019.119649 31791843

[78] Xie X Hu Y Ye T Chen Y Zhou L Li F Therapeutic vaccination against leukaemia via the sustained release of co-encapsulated anti-PD-1 and a leukaemia-associated antigen. Nat BioMed Eng (2020). 10.1038/s41551-020-00624-6 33046865

[79] Leleux J Roy K Micro and nanoparticle-based delivery systems for vaccine immunotherapy: an immunological and materials perspective. Adv Healthc Mater (2013) 2(1):72–94. 10.1002/adhm.201200268 23225517

[80] Cheng K Ding Y Zhao Y Ye S Zhao X Zhang Y Sequentially Responsive Therapeutic Peptide Assembling Nanoparticles for Dual-Targeted Cancer Immunotherapy. Nano Lett (2018) 18(5):3250–8. 10.1021/acs.nanolett.8b01071 29683683

[81] Ye X Liang X Chen Q Miao Q Chen X Zhang X Surgical Tumor-Derived Personalized Photothermal Vaccine Formulation for Cancer Immunotherapy. ACS Nano (2019) 13(3):2956–68. 10.1021/acsnano.8b07371 30789699

[82] Musetti S Huang L Nanoparticle-Mediated Remodeling of the Tumor Microenvironment to Enhance Immunotherapy. ACS Nano (2018) 12(12):11740–55. 10.1021/acsnano.8b05893 30508378

[83] Song C Phuengkham H Kim YS Dinh VV Lee I Shin IW Syringeable immunotherapeutic nanogel reshapes tumor microenvironment and prevents tumor metastasis and recurrence. Nat Commun (2019) 10(1):3745. 10.1038/s41467-019-11730-8 31431623 PMC6702226

[84] Xu J Wang H Xu L Chao Y Wang C Han X Nanovaccine based on a protein-delivering dendrimer for effective antigen cross-presentation and cancer immunotherapy. Biomaterials (2019) 207:1–9. 10.1016/j.biomaterials.2019.03.037 30947117

[85] Zhang Y Kurupati R Liu L Zhou XY Zhang G Hudaihed A Enhancing CD8(+) T Cell Fatty Acid Catabolism within a Metabolically Challenging Tumor Microenvironment Increases the Efficacy of Melanoma Immunotherapy. Cancer Cell (2017) 32(3):377–91.e9. 10.1016/j.ccell.2017.08.004 28898698 PMC5751418

[86] Altman BJ Stine ZE Dang CV From Krebs to clinic: glutamine metabolism to cancer therapy. Nat Rev Cancer (2016) 16(11):749. 10.1038/nrc.2016.114 28704361

[87] Scalise M Pochini L Galluccio M Console L Indiveri C Glutamine Transport and Mitochondrial Metabolism in Cancer Cell Growth. Front Oncol (2017) 7:306. 10.3389/fonc.2017.00306 29376023 PMC5770653

[88] Magill GB Myers WP Reilly HC Putnam RC Magill JW Sykes MP Pharmacological and initial therapeutic observations on 6-diazo-5-oxo-1-norleucine (DON) in human neoplastic disease. Cancer (1957) 10(6):1138–50. 10.1002/1097-0142(195711/12)10:6<1138::aid-cncr2820100608>3.0.co;2-k 13489662

[89] Li MC Whitmore WF Jr. Golbey R Grabstald H Effects of combined drug therapy on metastatic cancer of the testis. JAMA (1960) 174:1291–9. 10.1001/jama.1960.03030100059013 13761819

[90] A CLINICAL study of the comparative effect of nitrogen mustard and DON in patients with bronchogenic carcinoma, Hodgkin’s disease, lymphosarcoma, and melanoma. J Natl Cancer Inst (1959) 22(2):433–9. 13631504

[91] Leone RD Zhao L Englert JM Sun IM Oh MH Sun IH Glutamine blockade induces divergent metabolic programs to overcome tumor immune evasion. Science (2019) 366(6468):1013–21. 10.1126/science.aav2588 PMC7023461 31699883

[92] Brentjens RJ Davila ML Riviere I Park J Wang X Cowell LG CD19-targeted T cells rapidly induce molecular remissions in adults with chemotherapy-refractory acute lymphoblastic leukemia. Sci Transl Med (2013) 5(177):177ra38. 10.1126/scitranslmed.3005930 PMC3742551 23515080

[93] Cruz CR Micklethwaite KP Savoldo B Ramos CA Lam S Ku S Infusion of donor-derived CD19-redirected virus-specific T cells for B-cell malignancies relapsed after allogeneic stem cell transplant: a phase 1 study. Blood (2013) 122(17):2965–73. 10.1182/blood-2013-06-506741 PMC3811171 24030379

[94] Kochenderfer JN Somerville RPT Lu T Yang JC Sherry RM Feldman SA Long-Duration Complete Remissions of Diffuse Large B Cell Lymphoma after Anti-CD19 Chimeric Antigen Receptor T Cell Therapy. Mol Ther (2017) 25(10):2245–53. 10.1016/j.ymthe.2017.07.004 PMC5628864 28803861

[95] Maude SL Frey N Shaw PA Aplenc R Barrett DM Bunin NJ Chimeric antigen receptor T cells for sustained remissions in leukemia. N Engl J Med (2014) 371(16):1507–17. 10.1056/NEJMoa1407222 PMC4267531 25317870

[96] Sadelain M Brentjens R Riviere I The basic principles of chimeric antigen receptor design. Cancer Discov (2013) 3(4):388–98. 10.1158/2159-8290.CD-12-0548 PMC3667586 23550147

[97] Zhou G Levitsky H Towards curative cancer immunotherapy: overcoming posttherapy tumor escape. Clin Dev Immunol (2012) 2012:124187. 10.1155/2012/124187 22778760 PMC3386616

[98] Reinhard K Rengstl B Oehm P Michel K Billmeier A Hayduk N An RNA vaccine drives expansion and efficacy of claudin-CAR-T cells against solid tumors. Science (2020) 367(6476):446–53. 10.1126/science.aay5967 31896660

[99] Akahori Y Wang L Yoneyama M Seo N Okumura S Miyahara Y Antitumor activity of CAR-T cells targeting the intracellular oncoprotein WT1 can be enhanced by vaccination. Blood (2018) 132(11):1134–45. 10.1182/blood-2017-08-802926 PMC6148344 30045840

[100] Wang X Wong CW Urak R Mardiros A Budde LE Chang WC CMVpp65 Vaccine Enhances the Antitumor Efficacy of Adoptively Transferred CD19-Redirected CMV-Specific T Cells. Clin Cancer Res (2015) 21(13):2993–3002. 10.1158/1078-0432.CCR-14-2920 25838392 PMC4489991

[101] Wu M Zhang L Zhang H Ning J Tu S He Y CD19 chimeric antigen receptor-redirected T cells combined with epidermal growth factor receptor pathway substrate 8 peptide-derived dendritic cell vaccine in leukemia. Cytotherapy (2019) 21(6):659–70. 10.1016/j.jcyt.2019.03.313 31031152

[102] Slaney CY von Scheidt B Davenport AJ Beavis PA Westwood JA Mardiana S Dual-specific Chimeric Antigen Receptor T Cells and an Indirect Vaccine Eradicate a Variety of Large Solid Tumors in an Immunocompetent, Self-antigen Setting. Clin Cancer Res (2017) 23(10):2478–90. 10.1158/1078-0432.CCR-16-1860 PMC6369535 27965307

[103] Ueda Y Ogura M Miyakoshi S Suzuki T Heike Y Tagashira S Phase 1/2 study of the WT1 peptide cancer vaccine WT4869 in patients with myelodysplastic syndrome. Cancer Sci (2017) 108(12):2445–53. 10.1111/cas.13409 PMC5715294 28949050

[104] Rossig C Pule M Altvater B Saiagh S Wright G Ghorashian S Vaccination to improve the persistence of CD19CAR gene-modified T cells in relapsed pediatric acute lymphoblastic leukemia. Leukemia (2017) 31(5):1087–95. 10.1038/leu.2017.39 28126984

[105] Duraiswamy J Kaluza KM Freeman GJ Coukos G Dual blockade of PD-1 and CTLA-4 combined with tumor vaccine effectively restores T-cell rejection function in tumors. Cancer Res (2013) 73(12):3591–603. 10.1158/0008-5472.CAN-12-4100 PMC3686913 23633484

[106] Karyampudi L Lamichhane P Scheid AD Kalli KR Shreeder B Krempski JW Accumulation of memory precursor CD8 T cells in regressing tumors following combination therapy with vaccine and anti-PD-1 antibody. Cancer Res (2014) 74(11):2974–85. 10.1158/0008-5472.CAN-13-2564 PMC4313351 24728077

[107] Gibney GT Kudchadkar RR DeConti RC Thebeau MS Czupryn MP Tetteh L Safety, correlative markers, and clinical results of adjuvant nivolumab in combination with vaccine in resected high-risk metastatic melanoma. Clin Cancer Res (2015) 21(4):712–20. 10.1158/1078-0432.CCR-14-2468 PMC4620684 25524312

[108] Massarelli E William W Johnson F Kies M Ferrarotto R Guo M Combining Immune Checkpoint Blockade and Tumor-Specific Vaccine for Patients With Incurable Human Papillomavirus 16-Related Cancer: A Phase 2 Clinical Trial. JAMA Oncol (2019) 5(1):67–73. 10.1001/jamaoncol.2018.4051 30267032 PMC6439768

[109] Boussiotis VA Molecular and Biochemical Aspects of the PD-1 Checkpoint Pathway. N Engl J Med (2016) 375(18):1767–78. 10.1056/NEJMra1514296 PMC5575761 27806234

[110] Verma V Shrimali RK Ahmad S Dai W Wang H Lu S PD-1 blockade in subprimed CD8 cells induces dysfunctional PD-1(+)CD38(hi) cells and anti-PD-1 resistance. Nat Immunol (2019) 20(9):1231–43. 10.1038/s41590-019-0441-y PMC7472661 31358999

[111] Philip M Fairchild L Sun L Horste EL Camara S Shakiba M Chromatin states define tumour-specific T cell dysfunction and reprogramming. Nature (2017) 545(7655):452–6. 10.1038/nature22367 PMC5693219 28514453

[112] Ha D Tanaka A Kibayashi T Tanemura A Sugiyama D Wing JB Differential control of human Treg and effector T cells in tumor immunity by Fc-engineered anti-CTLA-4 antibody. Proc Natl Acad Sci U S A (2019) 116(2):609–18. 10.1073/pnas.1812186116 PMC6329979 30587582

[113] Brown MC Holl EK Boczkowski D Dobrikova E Mosaheb M Chandramohan V Cancer immunotherapy with recombinant poliovirus induces IFN-dominant activation of dendritic cells and tumor antigen-specific CTLs. Sci Transl Med (2017) 9(408):eaan4220. 10.1126/scitranslmed.aan4220 28931654 PMC6034685

[114] Kaufman HL Kohlhapp FJ Zloza A Oncolytic viruses: a new class of immunotherapy drugs. Nat Rev Drug Discov (2016) 15(9):660. 10.1038/nrd.2016.178 PMC7608450 30907381

[115] Di Paolo NC Miao EA Iwakura Y Murali-Krishna K Aderem A Flavell RA Virus binding to a plasma membrane receptor triggers interleukin-1 alpha-mediated proinflammatory macrophage response in vivo. Immunity (2009) 31(1):110–21. 10.1016/j.immuni.2009.04.015 PMC2759279 19576795

[116] Glorioso JC Cohen JB Goins WF Hall B Jackson JW Kohanbash G Oncolytic HSV Vectors and Anti-Tumor Immunity. Curr Issues Mol Biol (2020) 41:381–468. 10.21775/cimb.041.381 32938804

[117] Bridle BW Stephenson KB Boudreau JE Koshy S Kazdhan N Pullenayegum E Potentiating cancer immunotherapy using an oncolytic virus. Mol Ther (2010) 18(8):1430–9. 10.1038/mt.2010.98 PMC2927075 20551919

[118] Vestweber D How leukocytes cross the vascular endothelium. Nat Rev Immunol (2015) 15(11):692–704. 10.1038/nri3908 26471775

[119] Wakimoto H Ikeda K Abe T Ichikawa T Hochberg FH Ezekowitz RA The complement response against an oncolytic virus is species-specific in its activation pathways. Mol Ther (2002) 5(3):275–82. 10.1006/mthe.2002.0547 11863417

[120] Ilkow CS Marguerie M Batenchuk C Mayer J Ben Neriah D Cousineau S Reciprocal cellular cross-talk within the tumor microenvironment promotes oncolytic virus activity. Nat Med (2015) 21(5):530–6. 10.1038/nm.3848 25894825

[121] Gujar SA Marcato P Pan D Lee PW Reovirus virotherapy overrides tumor antigen presentation evasion and promotes protective antitumor immunity. Mol Cancer Ther (2010) 9(11):2924–33. 10.1158/1535-7163.MCT-10-0590 20978162

[122] Gujar SA Lee PW Oncolytic virus-mediated reversal of impaired tumor antigen presentation. Front Oncol (2014) 4:77. 10.3389/fonc.2014.00077 24782988 PMC3989761

[123] Koske I Rossler A Pipperger L Petersson M Barnstorf I Kimpel J Oncolytic virotherapy enhances the efficacy of a cancer vaccine by modulating the tumor microenvironment. Int J Cancer (2019) 145(7):1958–69. 10.1002/ijc.32325 PMC6767478 30972741

[124] McGray AJR Huang RY Battaglia S Eppolito C Miliotto A Stephenson KB Oncolytic Maraba virus armed with tumor antigen boosts vaccine priming and reveals diverse therapeutic response patterns when combined with checkpoint blockade in ovarian cancer. J Immunother Cancer (2019) 7(1):189. 10.1186/s40425-019-0641-x 31315674 PMC6637574

[125] Ylosmaki E Malorzo C Capasso C Honkasalo O Fusciello M Martins B Personalized Cancer Vaccine Platform for Clinically Relevant Oncolytic Enveloped Viruses. Mol Ther (2018) 26(9):2315–25. 10.1016/j.ymthe.2018.06.008 PMC6127500 30005865

[126] Ribas A Dummer R Puzanov I VanderWalde A Andtbacka RHI Michielin O Oncolytic Virotherapy Promotes Intratumoral T Cell Infiltration and Improves Anti-PD-1 Immunotherapy. Cell (2018) 174(4):1031–2. 10.1016/j.cell.2018.07.035 30096300

[127] Downs-Canner S Guo ZS Ravindranathan R Breitbach CJ O’Malley ME Jones HL Phase 1 Study of Intravenous Oncolytic Poxvirus (vvDD) in Patients With Advanced Solid Cancers. Mol Ther (2016) 24(8):1492–501. 10.1038/mt.2016.101 PMC5023393 27203445

[128] Mahalingam D Wilkinson GA Eng KH Fields P Raber P Moseley JL Pembrolizumab in Combination with the Oncolytic Virus Pelareorep and Chemotherapy in Patients with Advanced Pancreatic Adenocarcinoma: A Phase Ib Study. Clin Cancer Res (2020) 26(1):71–81. 10.1158/1078-0432.CCR-19-2078 31694832 PMC6942612

[129] Twumasi-Boateng K Pettigrew JL Kwok YYE Bell JC Nelson BH Oncolytic viruses as engineering platforms for combination immunotherapy. Nat Rev Cancer (2018) 18(7):419–32. 10.1038/s41568-018-0009-4 29695749

[130] James SR Link PA Karpf AR Epigenetic regulation of X-linked cancer/germline antigen genes by DNMT1 and DNMT3b. Oncogene (2006) 25(52):6975–85. 10.1038/sj.onc.1209678 16715135

[131] Roulois D Yau HL De Carvalho DD Pharmacological DNA demethylation: Implications for cancer immunotherapy. Oncoimmunology (2016) 5(3):e1090077. 10.1080/2162402X.2015.1090077 27141349 PMC4839380

[132] Sun T Li Y Yang W Wu H Li X Huang Y Histone deacetylase inhibition up-regulates MHC class I to facilitate cytotoxic T lymphocyte-mediated tumor cell killing in glioma cells. J Cancer (2019) 10(23):5638–45. 10.7150/jca.34471 PMC6843866 31737100

[133] Christiansen AJ West A Banks KM Haynes NM Teng MW Smyth MJ Eradication of solid tumors using histone deacetylase inhibitors combined with immune-stimulating antibodies. Proc Natl Acad Sci U S A (2011) 108(10):4141–6. 10.1073/pnas.1011037108 PMC3054015 21368108

[134] Gang AO Frosig TM Brimnes MK Lyngaa R Treppendahl MB Gronbaek K 5-Azacytidine treatment sensitizes tumor cells to T-cell mediated cytotoxicity and modulates NK cells in patients with myeloid malignancies. Blood Cancer J (2014) 4:e197. 10.1038/bcj.2014.14 24681961 PMC3972700

[135] Wang L Amoozgar Z Huang J Saleh MH Xing D Orsulic S Decitabine Enhances Lymphocyte Migration and Function and Synergizes with CTLA-4 Blockade in a Murine Ovarian Cancer Model. Cancer Immunol Res (2015) 3(9):1030–41. 10.1158/2326-6066.CIR-15-0073 26056145

[136] Shen L Ciesielski M Ramakrishnan S Miles KM Ellis L Sotomayor P Class I histone deacetylase inhibitor entinostat suppresses regulatory T cells and enhances immunotherapies in renal and prostate cancer models. PloS One (2012) 7(1):e30815. 10.1371/journal.pone.0030815 22303460 PMC3267747

[137] Ghoneim HE Fan Y Moustaki A Abdelsamed HA Dash P Dogra P De Novo Epigenetic Programs Inhibit PD-1 Blockade-Mediated T Cell Rejuvenation. Cell (2017) 170(1):142–57.e19. 10.1016/j.cell.2017.06.007 28648661 PMC5568784

[138] Cao K Wang G Li W Zhang L Wang R Huang Y Histone deacetylase inhibitors prevent activation-induced cell death and promote anti-tumor immunity. Oncogene (2015) 34(49):5960–70. 10.1038/onc.2015.46 PMC4672172 25745993

[139] Ehx G Fransolet G de Leval L D’Hondt S Lucas S Hannon M Azacytidine prevents experimental xenogeneic graft-versus-host disease without abrogating graft-versus-leukemia effects. Oncoimmunology (2017) 6(5):e1314425. 10.1080/2162402x.2017.1314425 28638744 PMC5467988

